# Residual Disparities in US Children Hospitalized Due to All-Cause Pneumonia Following Implementation of Childhood PCV13 in National Immunization Program

**DOI:** 10.3390/vaccines14080679

**Published:** 2026-08-06

**Authors:** Mark H. Rozenbaum, Ahuva Averin, Derek Weycker, Rotem Lapidot, Amanda Miles, Maria J. Tort, Jeffrey Vietri, Alexander Lonshteyn, Stephen I. Pelton

**Affiliations:** 1 Pfizer Inc., 2909 LD Capelle a/d Ijssel, The Netherlands; mark.rozenbaum@pfizer.com; 2 Avalere Health, Washington, DC 20005, USA; 3 Boston Medical Center, Boston, MA 02118, USAspelton@bu.edu (S.I.P.); 4 Rambam Health Care Campus, Israel Institute of Technology, Haifa 3109601, Israel; 5 Chobanian & Avedisian School of Medicine, Boston University, Boston, MA 02118, USA; 6 Pfizer Inc., Collegeville, PA 19426, USAmaria.tort@pfizer.com (M.J.T.);

**Keywords:** pneumonia, children, pneumococcal vaccines, comorbidity, socioeconomic factors

## Abstract

**Background/Objectives**: Pneumonia is a leading cause of hospitalization among children in the United States, with a disproportionate burden in certain racial, socioeconomic, and clinical subgroups. The 13-valent pneumococcal conjugate vaccine (PCV13), introduced in 2010, significantly reduced pneumococcal disease overall, but its effect on disparities in all-cause hospitalized pneumonia (AC-hPNA) remains unclear. This study evaluated changes in AC-hPNA rates by age, race, comorbidity profile, and household income (HHI) before and after PCV13 implementation. **Methods**: A retrospective cohort study using Optum’s de-identified Clinformatics^®^ DataMart (2007–2019) included children < 18 years. Rates (per 100,000 person-years) and incidence rate ratios were calculated for four periods: pre-PCV13 (2008–2009), peri-PCV13 (2010–2012), post-PCV13#1 (2013–2016), and post-PCV13#2 (2017–2019). Analyses were stratified by age group and, within each, by race, comorbidity profile (low, at-risk), and HHI. **Results**: Among 10.1 million children, AC-hPNA rates declined by 51–57% from pre-PCV13 to post-PCV13#2 across all age groups, with the largest reductions in children aged <2 years. Declines occurred across all race, comorbidity, and HHI subgroups; however, rates remained several-fold higher among at-risk children. Persistent disparities were observed among Black children < 2 years, children aged 2–5 years with HHI < $50,000, and children aged 6–17 years with HHI of $50,000–$99,999. **Conclusions**: Substantial reductions in rates of pediatric AC-hPNA were observed in the period subsequent to the introduction of routine PCV13, yet residual disparities persist among children with comorbidities and select racial and income groups. Targeted strategies are needed to address these gaps.

## 1. Introduction

*Streptococcus pneumoniae* (*S. pneumoniae*) is a leading cause of morbidity and mortality in children, particularly infants and those with underlying health conditions. It causes a spectrum of diseases, including non-bacteremic pneumonia (NBP), invasive pneumococcal disease (IPD)—a rare, but life-threatening, condition—and less severe infections such as otitis media [1]. *S. pneumoniae* is a major driver of community-acquired pneumonia (CAP), which is the second leading cause of childhood hospitalizations in the United States (US), after acute bronchiolitis [1,2,3]. In addition to young age and the presence of comorbidities, known risk factors for childhood pneumonia in the US include certain racial groups and low socioeconomic status [4,5].

The principal means of preventing pneumococcal disease is through immunization. The US Centers for Disease Control and Prevention (CDC) Advisory Committee on Immunization Practices (ACIP) currently recommends pneumococcal vaccination for all children under the age of five years and for older children aged ≤18 years with chronic medical conditions [6]. ACIP first recommended the use of the 7-valent pneumococcal conjugate vaccine (PCV; PCV7) in 2000 for children under 5 years old [7]. This guidance was superseded in 2010 by a recommendation for the 13-valent PCV (PCV13), which was also recommended for older children with certain underlying medical conditions [8]. More recently, 15-valent PCV (PCV15) and 20-valent PCV (PCV20) were added to the ACIP recommendations in 2022 and 2023, respectively [9,10]. Uptake of the PCV four-dose infant series increased from 54% to 80% between 2005 and 2008, and has remained between 80% and 85% from 2009 to 2023 [11].

The adoption of PCVs was highly effective at reducing the incidence as well as the racial, health, and socioeconomic disparities of IPD among the US population [12,13,14,15,16]. Although the data are limited, studies on all-cause pneumonia following pediatric PCV13 introduction suggest that the number of hospitalizations decreased across age groups. Wiese et al. found that the pneumonia hospitalization rates in Tennessee fell 49% from the pre-PCV7 to post-PCV13 period, with racial disparities no longer persisting post-PCV13 [13]. Similarly, Hu et al. reported a 25% decrease in all-cause hospitalized pneumonia among children from the late PCV7 to early PCV13 period based on claims data [14]. The aim of this study was to evaluate the impact of the 2010 ACIP recommendations for childhood PCV13 immunization on rates of pediatric all-cause hospitalized pneumonia (AC-hPNA) by race, household income (HHI), and comorbidity profile.

## 2. Materials and Methods

### 2.1. Study Design and Data Source

A retrospective observational cohort study was conducted using claims data from Optum’s Clinformatics^®^ Data Mart Database (CDM) [17]. The data spanned January 2008 through December 2019, encompassing the two years before and the decade following the implementation of the 2010 ACIP recommendation for childhood PCV13 immunization. Optum’s de-identified CDM is a national database comprising insurer-provided medical (i.e., facility and professional service) claims, outpatient pharmacy claims, and enrollment information. The data source includes approximately 2 million commercially insured pediatric lives annually across all 50 US states. Data available from medical claims include diagnoses, procedures, dates and places of service, and provider category. Data available from pharmacy claims include the prescription product, date of prescription, days supplied, and quantity supplied. All healthcare claims are verified, adjudicated, adjusted, and de-identified by Optum prior to their inclusion in the database. CDM also provides access to demographic and eligibility information, such as age, sex, race, and HHI. Variables for race and HHI are based on a mix of individual-level and ecological-level data.

### 2.2. Study Population

The study population comprised US children aged <18 years old with ≥1 day of healthcare coverage during the period spanning 1 January 2008, through 31 December 2019. For children aged ≥2 years, the first day of healthcare coverage during the study period (2008–2019) had to be preceded by ≥1 year of continuous healthcare coverage. Because children aged <2 years may be newly enrolled from birth and may lack a full year of coverage, they were not subject to this requirement. For children aged <2 years, the index date was the first day of coverage; for children aged ≥2 years, it was the day after the initial 1-year continuous enrollment period ended. For children who had multiple unique qualifying periods of healthcare coverage, data from each period were considered independently in analyses.

### 2.3. Study Variables

The study population was characterized at baseline based on age (<2 years, 2–5 years, 6–17 years), sex, race (Asian, Black, Hispanic, White, unknown/missing), comorbidity profile (low-risk, at-risk), and HHI. Comorbid conditions of interest included those that increase the risk of pneumococcal disease [8,18]. Children were therefore considered “at-risk” if they had ≥1 risk condition (i.e., asplenia, asthma, blood cell disorders, cerebrospinal fluid leak, cochlear implant, congenital immunodeficiency, diabetes, heart disease, human immunodeficiency virus, immunosuppressant use, liver disease, lung disease, malignancy, nephrotic syndrome, neuromuscular disorders, organ transplant, prematurity, renal disease, and trisomy 21) and children without evidence of risk conditions were considered “low-risk”; the codes that were employed to identify risk conditions in claims data are available upon request. Annual HHI was categorized according to the following strata: <$50,000, $50,000–$99,000, ≥$100,000 (herein referred to as “low”, “middle”, and “high”, respectively), or unknown/missing. Vaccination status could not be reliably ascertained in the study database for two reasons: (1) the inability of claims data to capture vaccines administered through the Vaccines for Children (VFC) program, which provides vaccines at no cost to approximately 50% of US children—including some commercially insured children—and does not generate a vaccine product claim [19] and (2) the high rates of plan turnover in commercial health plans, which preclude the construction of complete longitudinal vaccination histories.

Episodes of AC-hPNA were identified based on inpatient admissions with a diagnosis of pneumonia in the principal position or with a diagnosis of respiratory failure in the principal position and pneumonia in the secondary position. Diagnosis codes employed are available in Appendix A. Inpatient admissions were assessed for each month beginning on the index date and ending on the last date of health plan enrollment or the last date of the study period, whichever occurred first. All qualifying hospitalizations that occurred within 30 days of each other were combined into one episode.

### 2.4. Statistical Analysis

Baseline characteristics were summarized via percentages. Imputation for missing data and statistical weighting to adjust the sample to population characteristics were not performed. Rates of AC-hPNA were estimated a priori during four periods: pre-PCV13 (2008–2009), peri-PCV13 (2010–2012), post-PCV13#1 (2013–2016), and post-PCV13#2 (2017–2019). The post-PCV13 era was divided into three periods—a ‘peri’ PCV introduction (2010–2012), an early PCV13 period (2013–2016), and a later PCV13 period (2017–2019)—to evaluate temporal trends in rates of pediatric AC-hPNA across the 10-year period following the approval and implementation of PCV13. Rates were estimated by age as well as within age-specific subgroups defined according to race, comorbidity profile, and HHI. Rates were reported per 100,000 person-years and were adjusted for differential follow-up using a population-based approach (i.e., total number of episodes divided by total number of person-days of observation, multiplied by 365.25); techniques of nonparametric bootstrapping were used to estimate 95% confidence intervals (CIs). Poisson regression models were employed to estimate differences in disease incidence (i.e., incidence rate ratios [IRRs]) between periods and subgroups of interest; regression models were not adjusted for baseline covariates.

### 2.5. Ethics Statement

Ethical review and approval were waived for this study due to the use of retrospective, de-identified healthcare claims data that do not constitute human subject research, as defined under 45 CFR 46.102.

## 3. Results

### 3.1. Baseline Characteristics

The study population included 9.4 million children aged <18 years, contributing 10.1 million unique qualifying periods; 26% were aged <2 years, 14% were aged 2–5 years, and 60% were aged 6–17 years (Table 1). Across age groups, most children were White (49–62%), followed by Hispanic (9–11%), Black (6–8%), and Asian (5–6%); race was missing for 15–31% of children. The large majority of children were low-risk (95–98%), with 2–5% designated as at-risk. Annual HHI was <$50,000 for 9–14% of children, $50,000-$99,999 for 17–20%, and >$100,000 for 29–37%; HHI was missing for 29–45% of children.

### 3.2. Rates and IRR of AC-hPNA by Age

The incidence of AC-hPNA declined from the pre-PCV13 to post-PCV13#2 period across all age groups (Table 2). The highest incidence during both periods was observed among children aged <2 years, with rates of AC-hPNA per 100,000 person-years (95% CI) decreasing from 410.2 (391.7–429.5) in the pre-PCV13 period to 177.0 (165.4–189.4) in the post-PCV13#2 period. For children aged 2–5 years, rates declined from 265.6 (254.9–276.7) to 129.0 (122.3–136.0), and for those aged 6–17 years, from 63.0 (60.1–65.9) to 30.0 (28.3–31.9).

The youngest children aged <2 years experienced a nominally greater reduction in the incidence of AC-hPNA from the pre-PCV13 to post-PCV13#2 periods, with an IRR (95% CI) of 0.43 (0.40–0.47), corresponding to a relative reduction of 57%. The relative reduction for children aged 2–5 years and aged 6–17 years was 51% and 52%, respectively.

### 3.3. Age-Specific Rates and IRR of AC-hPNA Comparing Pre-PCV13 Versus Post-PCV13#2 by Race, Comorbidity Profile, and HHI

During the pre-PCV13 period, the incidence of AC-hPNA was consistently several-fold higher among children with at-risk medical conditions compared to low-risk children across all age groups (Table 3). Smaller differences in AC-hPNA incidence were also observed by race and HHI. Among children aged <2 and 2–5 years, rates were higher for Black compared to White and Asian groups, and for low-income versus middle- and high-income groups. Additionally, among children aged 6–17 years, AC-hPNA rates were higher for low-income compared to high-income children.

The incidence of AC-hPNA generally decreased over the four study periods from pre-PCV13 to post-PCV13#2 across all age groups and within each race, comorbidity, and HHI subgroup (Supplementary Materials, Tables S1–S3). A decline in incidence rates was observed during the post-PCV13#2 period (vs. pre-PCV13) for all race, comorbidity profile, and HHI groups, and the IRRs ranged from 0.37 (0.27–0.49) to 0.63 (0.49–0.81), corresponding to relative reductions of 37% to 63% (Table 3). During the post-PCV13#2 period, the incidence of AC-hPNA remained highest among at-risk children. The age-specific incidence rates among children for whom race and HHI were unknown/missing were similar to the age-specific overall incidence rates.

### 3.4. Age-Specific IRR of AC-hPNA in Post-PCV13#2 Period by Race, Comorbidity Profile, and HHI

Despite overall declines in AC-hPNA incidence after PCV13 introduction, disparities persisted during the post-PCV13#2 period among children with at-risk medical conditions (Table 4). AC-hPNA incidence among the at-risk group was 5.04 (4.07–6.24), 5.64 (4.84–6.57), and 8.84 (7.75–10.08) times higher compared to the low-risk group among children aged <2, 2–5, and 6–17 years, respectively. Smaller disparities were observed among Black children aged <2 years (IRR vs. White: 1.43 [1.05–1.97]), low-income children aged 2–5 years (IRR vs. high-income: 1.24 [1.04–1.49]), and middle-income children aged 6–17 years (IRR vs. high-income: 1.21 [1.04–1.41]).

## 4. Discussion

This study, based on data from over 10 million US children, estimated AC-hPNA rates by age and, within age groups, by race, comorbidity profile, and HHI before and after the 2010 ACIP recommendation for routine pediatric PCV13. From the pre-PCV13 to post-PCV13#2 periods, AC-hPNA rates declined by 37–63% across age and demographic subgroups, with the highest incidence and greatest proportional reduction among children aged <2 years. Despite these substantial declines following the introduction of pediatric PCV13, rates remained several-fold higher among at-risk children. In the post-PCV13#2 period, smaller disparities persisted among Black children aged <2 years, low-income children aged 2–5 years, and middle-income children aged 6–17 years.

Our findings align with prior US studies showing marked reductions in pediatric pneumonia hospitalizations following PCV introduction. Hu et al. (2023) reported declines of 47–64% from the pre-PCV7 to late PCV13 periods and 25–31% between the early and late PCV13 periods, with the largest reductions among children aged <2 years [14]. Other US studies [13,20], as well as studies from England [21], Sweden [22], and South Africa [23], have also reported reductions in all-cause pneumonia hospitalizations among children, with the largest declines generally seen in the youngest age groups [21,22,23].

In comparison, previous findings on the impact of PCV immunization on racial, health, and socioeconomic disparities in all-cause pneumonia among children are notably more limited. A US-based study by Wiese et al. analyzed racial disparities in pneumonia hospitalizations among Tennessee residents aged <18 years [13]. The authors found that, while rates were higher among Black children compared to White children during the pre-PCV7 period (1998–1999), rates declined for both groups with only minimal residual racial differences observed during the post-PCV13 period (2011–2013). In the present study, AC-hPNA incidence rates during the pre-PCV13 period (2008–2009) were higher among Black compared to White children, but this difference was observed only among those aged ≤5 years (Supplementary Materials, Tables S1 and S2). Unlike Wiese et al., we found that the racial disparity persisted among children aged 2–5 years during the peri-PCV13 period (2010–2012) [13]. However, direct comparisons between studies should be made with caution due to differences in methodologies and study periods.

Finally, the present study found that the incidence of AC-hPNA was consistently several-fold higher among children with comorbidities, and similar findings have been reported in Sweden [22] and South Africa [23]. Regarding SES, evidence from England [21] showed that incidence of hospitalized pneumonia from 2003 to 2019 was lowest among children from the least-deprived areas and increased with greater socioeconomic deprivation. Similarly, in the present study, disparities in AC-hPNA incidence were observed among low-income children aged <6 years.

The present study has a number of limitations that should be noted. Firstly, we recognize that declines in AC-hPNA rates are multi-factorial; the observed reductions may not be entirely attributable to vaccination and may also be a result of changes to practice patterns and the provision of expanded in-home medical services, in addition to the introduction of PCV13. Moreover, because our study measured rates of all-cause hPNA, it is not possible to ascertain the proportion of the observed reduction in disease that is attributable to a reduction in pneumococcal pneumonia (vs. non-pneumococcal pneumonia). Secondly, while Optum CDM provides data on a large sample of patients across demographic profiles, providers, and geographic regions, the study results may not be generalizable to other populations or settings. For example, the study database excludes individuals who are uninsured or covered by other types of health insurance (e.g., Medicaid). In addition, analyses were constrained by the available variables, including limited race categorization, and the presence of missing or unknown data. Notably, however, observed incidence rates among children for whom the race and HHI were unknown/missing were comparable to those of the overall study population, suggesting that children with unknown/missing data are not systematically different from their counterparts. Thirdly, even though similar operational algorithms have been implemented in previous research [24,25,26,27], the categorization of patients based on their comorbidity profile and the identification of pneumonia episodes may have resulted in some misclassification bias. Finally, insurance claims data primarily rely on computational methods (e.g., algorithms) and indirect sources such as geographic proxies to derive race, ethnicity, and HHI variables. The race and household income variables in the Optum CDM are based on a mix of individual-level and ecological-level data and, to our knowledge, have not been evaluated against a gold standard. Additionally, the lack of standardized definitions and frequent missing data can compromise the validity and generalizability of analyses involving race/ethnicity and HHI. Finally, healthcare claim data do not allow for accurate estimation of the uptake of pediatric vaccines, and we were therefore unable to explicitly estimate vaccine effectiveness or conduct stratified analyses based on receipt (yes/no) of PCV13.

## 5. Conclusions

Following the 2010 ACIP recommendation for PCV13 childhood immunization in the US, pediatric AC-hPNA rates declined markedly for all age groups and racial, comorbidity, and HHI subgroups. However, residual disparities remained evident among younger Black and low-income children, and were particularly pronounced in children with comorbidities. These findings underscore the continuing importance of childhood immunization strategies to reduce the burden of all-cause pneumonia among vulnerable populations. Closing these residual gaps may require targeted strategies, such as EHR-based reminders and recall systems to support PCV series completion in children with risk conditions, community partnerships to help reach populations with persistently high disease burden, and efforts to reduce structural barriers such as transportation and clinic access. Future research could explore these approaches, alongside the continued introduction of higher-valency PCVs, as potential avenues for reducing pneumococcal disease burden among pediatric populations.

## Figures and Tables

**Table 1:** Baseline characteristics of study population.

			All (N = 10,059,240)	Age < 2 (N = 2,575,181)	Age 2–5 (N = 1,434,456)	Age 6–17 (N = 6,049,603)
Age group (years), %						
	<2		25.6%	100.0%	--	--
	2–5		14.3%	--	100.0%	--
	6–17		60.1%	--	--	100.0%
Sex, %						
	Female		48.9%	48.6%	48.8%	49.0%
	Male		51.1%	51.3%	51.2%	51.0%
Race, %						
	Asian		5.1%	5.5%	6.2%	4.6%
	Black		7.6%	5.8%	7.2%	8.4%
	Hispanic		10.1%	8.5%	10.2%	10.8%
	White		57.9%	48.9%	58.2%	61.6%
	Unknown/missing		19.4%	31.3%	18.2%	14.6%
Comorbidity profile, %						
	Low-risk		96.0%	97.7%	94.6%	95.5%
	At-risk		4.0%	2.3%	5.4%	4.5%
		Asplenia	0.0%	0.0%	0.0%	0.0%
		Asthma	2.6%	0.0%	4.0%	3.3%
		Blood cell disorders	0.0%	0.0%	0.1%	0.0%
		Cerebrospinal fluid leak	0.0%	0.0%	0.0%	0.0%
		Cochlear implant	0.0%	0.0%	0.0%	0.0%
		Congenital immunodeficiency	0.1%	0.0%	0.2%	0.1%
		Diabetes	0.2%	0.0%	0.1%	0.3%
		Heart disease	0.2%	0.2%	0.2%	0.1%
		Human immunodeficiency virus	0.0%	0.0%	0.0%	0.0%
		Immunosuppressant use	0.1%	0.0%	0.3%	0.1%
		Liver disease	0.0%	0.0%	0.0%	0.0%
		Lung disease	0.1%	0.0%	0.2%	0.1%
		Malignancy	0.1%	0.0%	0.2%	0.2%
		Nephrotic syndrome	0.0%	0.0%	0.0%	0.0%
		Neuromuscular disorders	0.1%	0.2%	0.1%	0.1%
		Organ transplant	0.0%	0.0%	0.0%	0.0%
		Prematurity	0.5%	1.8%	0.1%	0.0%
		Renal disease	0.0%	0.0%	0.0%	0.0%
		Trisomy 21	0.1%	0.1%	0.1%	0.1%
Household income (annual), %						
	<$50,000		12.0%	8.9%	10.4%	13.8%
	$50,000-$99,999		19.1%	16.5%	18.7%	20.3%
	≥$100,000		34.8%	29.3%	36.3%	36.7%
	Unknown/missing		34.1%	45.3%	34.6%	29.2%

**Table 2:** Rates of AC-hPNA and disease incidence rate ratios by age.

			Pre-PCV13 (2008–2009)	Peri-PCV13 (2010–2012)	Post-PCV13#1 (2013–2016)	Post-PCV13#2 (2017–2019)
Age group (years)						
	<2					
		Rate/100K PY (95% CI)	410.2 (391.7–429.5)	332.0 (317.0–347.7)	215.1 (204.6–226.1)	177.0 (165.4–189.4)
		IRR (95% CI)	referent	0.81 (0.76–0.86)	0.52 (0.49–0.56)	0.43 (0.40–0.47)
		No. of PY	442,506	539,785	719,195	470,647
	2–5					
		Rate/100K PY (95% CI)	265.6 (254.9–276.7)	233.9 (225.2–243.0)	176.1 (169.1–183.4)	129.0 (122.3–136.0)
		IRR (95% CI)	referent	0.88 (0.83–0.93)	0.66 (0.63–0.70)	0.49 (0.45–0.52)
		No. of PY	861,938	1,135,014	1,329,803	1,045,302
	6–17					
		Rate/100K PY (95% CI)	63.0 (60.1–65.9)	54.4 (52.1–56.7)	39.6 (37.9–41.5)	30.0 (28.3–31.9)
		IRR (95% CI)	referent	0.86 (0.81–0.92)	0.63 (0.59–0.67)	0.48 (0.44–0.51)
		No. of PY	2,860,143	3,892,463	4,678,346	3,591,493

AC-hPNA: all-cause hospitalized pneumonia; CI: confidence interval; IRR: incidence rate ratio; PCV13: 13-valent pneumococcal conjugate vaccine; PY: person-year.

**Table 3:** Age-specific rates of AC-hPNA and disease incidence rate ratios by race, comorbidity profile, and household income.

			Age < 2 Years		Age 2–5 Years		Age 6–17 Years	
			Pre-PCV13 (2008–2009)	Post-PCV13#2 (2017–2019)	Pre-PCV13 (2008–2009)	Post-PCV13#2 (2017–2019)	Pre-PCV13 (2008–2009)	Post-PCV13#2 (2017–2019)
Race								
	Asian							
		Rate/100K PY (95% CI)	313.4 (248.4–395.5)	174.3 (125.1–242.7)	225.5 (186.4–272.7)	115.7 (94.2–142.0)	58.2 (46.0–73.7)	25.0 (19.3–32.4)
		IRR (95% CI)	referent	0.56 (0.37–0.83)	referent	0.51 (0.39–0.68)	referent	0.43 (0.30–0.61)
		No. of PY	22,654	20,083	47,016	78,681	118,538	227,775
	Black							
		Rate/100K PY (95% CI)	572.2 (490.1–668.1)	260.7 (194.0–350.3)	329.3 (286.5–378.5)	126.8 (102.0–157.6)	72.8 (62.9–84.3)	28.7 (22.9–35.9)
		IRR (95% CI)	referent	0.46 (0.33–0.64)	referent	0.39 (0.30–0.50)	referent	0.39 (0.30–0.51)
		No. of PY	27,962	16,877	60,131	63,888	247,181	265,211
	Hispanic							
		Rate/100K PY (95% CI)	464.8 (399.5–540.6)	174.7 (132.4–230.5)	304.9 (268.9–345.6)	138.2 (118.7–160.9)	57.5 (49.2–67.2)	29.1 (24.5–34.5)
		IRR (95% CI)	referent	0.38 (0.27–0.52)	referent	0.45 (0.37–0.55)	referent	0.51 (0.40–0.64)
		No. of PY	36,147	28,623	80,030	120,107	274,700	454,158
	White							
		Rate/100K PY (95% CI)	407.8 (382.3–435.0)	181.7 (162.3–203.4)	256.0 (242.3–270.5)	126.1 (117.7–135.1)	63.0 (59.4–66.9)	30.8 (28.6–33.1)
		IRR (95% CI)	referent	0.45 (0.39–0.51)	referent	0.49 (0.45–0.54)	referent	0.49 (0.44–0.54)
		No. of PY	225,613	165,652	496,100	640,991	1,741,493	2,364,135
	Unknown/missing							
		Rate/100K PY (95% CI)	381.2 (349.0–416.2)	168.3 (152.7–185.6)	263.6 (240.9–288.5)	142.6 (124.2–163.7)	61.9 (55.2–69.4)	31.0 (25.2–38.3)
		IRR (95% CI)	referent	0.44 (0.39–0.50)	referent	0.54 (0.46–0.64)	referent	0.50 (0.39–0.64)
		No. of PY	130,130	239,412	178,661	141,636	478,230	280,214
Comorbidity profile								
	Low-risk							
		Rate/100K PY (95% CI)	385.5 (367.4–404.5)	160.6 (149.5–172.7)	227.4 (217.4–238.0)	113.8 (107.5–120.6)	46.9 (44.4–49.6)	22.3 (20.8–24.0)
		IRR (95% CI)	referent	0.42 (0.38–0.45)	referent	0.50 (0.47–0.54)	referent	0.48 (0.44–0.52)
		No. of PY	430,394	458,791	825,731	1,015,417	2,731,647	3,433,479
	At-risk							
		Rate/100K PY (95% CI)	1287.9 (1100.9–1506.8)	809.7 (662.9–989.0)	1135.1 (1030.5–1250.4)	642.5 (557.7–740.1)	403.9 (370.6–440.2)	197.5 (176.7–220.6)
		IRR (95% CI)	referent	0.63 (0.49–0.81)	referent	0.57 (0.48–0.67)	referent	0.49 (0.42–0.56)
		No. of PY	12,112	11,856	36,207	29,885	128,496	158,014
Household income								
	<$50,000							
		Rate/100K PY (95% CI)	629.5 (551.6–718.4)	231.1 (177.4–301.0)	372.9 (331.0–420.1)	151.1 (128.7–177.3)	73.3 (65.1–82.5)	30.2 (25.4–35.8)
		IRR (95% CI)	referent	0.37 (0.27–0.49)	referent	0.41 (0.33–0.49)	referent	0.41 (0.33–0.51)
		No. of PY	34,948	23,799	72,407	99,282	371,185	431,054
$50,000–$99,999								
		Rate/100K PY (95% CI)	472.5 (425.3–524.9)	189.8 (155.0–232.3)	283.8 (258.3–311.8)	131.1 (116.5–147.5)	64.4 (58.2–71.2)	34.9 (30.9–39.4)
		IRR (95% CI)	referent	0.40 (0.32–0.50)	referent	0.46 (0.40–0.54)	referent	0.54 (0.46–0.63)
		No. of PY	73,442	49,537	152,920	210,519	591,908	733,941
	≥$100,000							
		Rate/100K PY (95% CI)	331.1 (303.0–361.8)	183.0 (156.9–213.5)	228.2 (212.8–244.7)	121.6 (111.6–132.5)	58.5 (54.1–63.3)	28.9 (26.4–31.6)
		IRR (95% CI)	referent	0.55 (0.46–0.66)	referent	0.53 (0.48–0.60)	referent	0.49 (0.44–0.56)
		No. of PY	147,381	88,525	343,583	425,943	1,076,923	1,663,471
	Unknown/missing							
		Rate/100K PY (95% CI)	407.0 (379.1–437.0)	169.0 (155.2–184.2)	273.4 (255.1–293.0)	130.5 (118.4–143.9)	63.2 (57.9–68.8)	27.9 (24.4–31.9)
		IRR (95% CI)	referent	0.42 (0.37–0.46)	referent	0.48 (0.42–0.54)	referent	0.44 (0.38–0.52)
		No. of PY	186,736	308,786	293,028	309,559	820,127	763,027

AC-hPNA: all-cause hospitalized pneumonia; CI: confidence interval; IRR: incidence rate ratio; PCV13: 13-valent pneumococcal conjugate vaccine; PY: person-year.

**Table 4:** Age-specific incidence rate ratios of AC-hPNA in post-PCV13#2 period by race, comorbidity profile, and household income.

		Age < 2 Years	Age 2–5 Years	Age 6–17 Years
Race, IRR (95% CI)				
	Asian	0.96 (0.68–1.36)	0.92 (0.74–1.14)	0.81 (0.62–1.07)
	Black	1.43 (1.05–1.97)	1.01 (0.80–1.26)	0.93 (0.74–1.18)
	Hispanic	0.96 (0.71–1.30)	1.10 (0.93–1.30)	0.95 (0.79–1.14)
	White	referent	referent	referent
	Unknown/missing	0.93 (0.80–1.08)	1.13 (0.97–1.32)	1.01 (0.81–1.26)
Comorbidity profile, IRR (95% CI)				
	Low-risk	referent	referent	referent
	At-risk	5.04 (4.07–6.24)	5.64 (4.84–6.57)	8.84 (7.75–10.08)
Household income, IRR (95% CI)				
	<$50,000	1.26 (0.93–1.71)	1.24 (1.04–1.49)	1.05 (0.86–1.27)
	$50,000-$99,999	1.04 (0.80–1.34)	1.08 (0.93–1.25)	1.21 (1.04–1.41)
	≥$100,000	referent	referent	referent
	Unknown/missing	0.92 (0.77–1.10)	1.07 (0.94–1.22)	0.97 (0.82–1.14)

AC-hPNA: all-cause hospitalized pneumonia; CI: confidence interval; IRR: incidence rate ratio; PCV13: 13-valent pneumococcal conjugate vaccine; PY: person-year.

## Data Availability

The study data sources are proprietary and were provided by a third-party vendor, and the authors do not have permission to disseminate the data without vendor approval.

## Supplementary Materials

The following supporting information can be downloaded at: https://www.mdpi.com/article/10.3390/vaccines14080679/s1, Table S1: Rates of AC-hPNA and disease incidence rate ratios among children aged <2 years by race, comorbidity profile, and household income. Table S2: Rates of AC-hPNA and disease incidence rate ratios among children aged 2–5 years by race, comorbidity, and household income. Table S3. Rates of AC-hPNA and disease incidence rate ratios among children aged 6–17 years by race, comorbidity profile, and household income.

## Abbreviations

The following abbreviations are used in this manuscript:

AC-hPNA	All-cause hospitalized pneumonia
ACIP	Advisory Committee on Immunization Practices
CAP	Community-acquired pneumonia
CDC	Centers for Disease Control and Prevention
CMD	Clinformatics^®^ Data Mart Database
HHI	Household income
IPD	Invasive pneumococcal disease
IRR	Incidence rate ratios
NBP	Non-bacteremic pneumonia
PCV7	7-valent pneumococcal conjugate vaccine
PCV13	13-valent pneumococcal conjugate vaccine
PCV15	15-valent pneumococcal conjugate vaccine
PCV20	20-valent pneumococcal conjugate vaccine
SES	Socioeconomic status

## Appendix A

**Table A1:** Operational algorithms for identifying episodes of AC-hPNA †.

Outcome	ICD-9-CM Dx	ICD-10-CM Dx	Algorithms
Invasive pneumococcal disease	Invasive disease: 038.0, 038.9, 041.00, 320.9, 322.9, 421.x, 711.0x, 511.89, 513.x, 730.0x, 730.2x, 771.81, 771.83, 785.52, 790.7, 995.91, 995.92 AND Pathogen: 041.2 OR Pathogen-specific invasive disease: 038.2, 320.1	Invasive disease: A40.9, A41.9, B95.5, G00.9, G03.9, G04.2, I33.0, I33.9, I39, J85.1, J85.2, J95.851, M00.***, M46.2 *, M86.0 **, M86.1 **, M86.2 **, P36.9, R65.2 *, R78.81 AND Pathogen: B95.3 OR Pathogen-specific invasive disease: A40.3, G00.1	Acute-care inpatient admission with: - Diagnosis code for invasive disease in principal position AND diagnosis code for pathogen in secondary position; OR - Diagnosis code for pathogen in principal position AND diagnosis code for invasive disease in secondary position; OR - Diagnosis code for pathogen-specific invasive disease in principal position
All-cause non-bacteremic pneumonia	Pneumonia: 115.05, 115.15, 115.95, 480.x–486, 487.0, 488.01, 488.11, 488.81, 510.x, 511.1, 511.9, 516.30, 516.33 OR ‘Respiratory failure: 518.81, 518.83, 518.84, 799.1 AND Pneumonia (above)	Pneumonia: A22.1, A37.01, A37.11, A37.81, A37.91, A48.1, B01.2, B05.2, B06.81, B25.0, B44.0, B77.81, J09.X1, J10.0 *, J11.0 *, J12. **, J13, J14, J15. ***, J16. *, J17, J18.0, J18.1, J18.8, J18.9, J86. *, J90, J91.8 OR Respiratory failure: J96.00, J96.1 *, J96.20, J96.90, R09.2 AND Pneumonia (above)	Acute-care inpatient admission with: - Diagnosis code for pneumonia in principal position; OR - Diagnosis code for respiratory failure in principal position AND diagnosis code for pneumonia in secondary position Note: hospitalizations with evidence of IPD and PNA were classified as IPD and excluded.

†: All qualifying encounters (i.e., those with a diagnosis code of interest) occurring within 30 days of each other were considered part of the same episode (i.e., qualifying encounters can be separated by no more than 30 days). Multiple episodes per patient were identified. *: one additional character unspecified; **: two additional characters unspecified; ***: three additional characters unspecified.
