# Supplementary material for: Residual Disparities in US Children Hospitalized Due to All-Cause Pneumonia Following Implementation of Childhood PCV13 in National Immunization Program
Source: Vaccines (Basel). 2026 Aug 6;14(8):679. doi: 10.3390/vaccines14080679 (PMC13517589; doi:10.3390/vaccines14080679)
Supplement: Supplementary file 1 [file vaccines-14-00679-s001.zip › vaccines-4395571-supplementary.pdf]

vaccines

MDPI

Supplementary materials

Supplement - Table S1. Rates of AC-hPNA and disease incidence rate ratios among children aged <2 years by race, comorbidity profile, and household income

	Pre-PCV13 (2008-2009)	Peri-PCV13 (2010-2012)	Post-PCV13#1 (2013-2016)	Post-PCV13#2 (2017-2019)
Race				
Asian				
Rate/100K PY (95% CI)	313.4 (248.4-395.5)	242.6 (195.7-300.9)	168.1 (137.7-205.4)	174.3 (125.1-242.7)
IRR (95% CI)	referent	0.77 (0.56-1.06)	0.54 (0.39-0.73)	0.56 (0.37-0.83)
No. of PY	22,654	34,207	57,093	20,083
Black				
Rate/100K PY (95% CI)	572.2 (490.1-668.1)	406.8 (345.9-478.4)	276.8 (233.0-328.7)	260.7 (194.0-350.3)
IRR (95% CI)	referent	0.71 (0.57-0.89)	0.48 (0.38-0.61)	0.46 (0.33-0.64)
No. of PY	27,962	35,890	46,974	16,877
Hispanic				
Rate/100K PY (95% CI)	464.8 (399.5-540.6)	435.0 (379.4-498.6)	250.2 (217.4-288.0)	174.7 (132.4-230.5)
IRR (95% CI)	referent	0.94 (0.76-1.15)	0.54 (0.44-0.66)	0.38 (0.27-0.52)
No. of PY	36,147	47,360	77,536	28,623
White				
Rate/100K PY (95% CI)	407.8 (382.3-435.0)	336.2 (316.7-356.9)	218.4 (205.2-232.4)	181.7 (162.3-203.4)
IRR (95% CI)	referent	0.82 (0.75-0.90)	0.54 (0.49-0.59)	0.45 (0.39-0.51)
No. of PY	225,613	320,355	453,847	165,652
Unknown				
Rate/100K PY (95% CI)	381.2 (349.0-416.2)	274.6 (244.2-308.7)	162.4 (137.3-192.1)	168.3 (152.7-185.6)
IRR (95% CI)	referent	0.72 (0.62-0.83)	0.43 (0.35-0.52)	0.44 (0.39-0.50)
No. of PY	130,130	101,973	83,746	239,412
Comorbidity profile				
Low-risk				
Rate/100K PY (95% CI)	385.5 (367.4-404.5)	306.9 (292.3-322.3)	197.6 (187.5-208.3)	160.6 (149.5-172.7)
IRR (95% CI)	referent	0.80 (0.74-0.85)	0.51 (0.48-0.55)	0.42 (0.38-0.45)
No. of PY	430,394	524,939	700,741	458,791
At-risk				
Rate/100K PY (95% CI)	1,287.9 (1,100.9-1,506.8)	1,219.2 (1,053.9-1,410.4)	877.9 (752.6-1,024.1)	809.7 (662.9-989.0)
IRR (95% CI)	referent	0.95 (0.76-1.17)	0.68 (0.55-0.85)	0.63 (0.49-0.81)
No. of PY	12,112	14,846	18,453	11,856

AC-hPNE: all-cause hospitalized pneumonia; CI: confidence interval; IRR: incidence rate ratio; PCV13: 13-valent pneumococcal conjugate vaccine; PY: person year

Supplement - Table S1. Rates of AC-hPNA and disease incidence rate ratios among children aged <2 years by race, comorbidity profile, and household income (cont.)

	Pre-PCV13 (2008-2009)	Peri-PCV13 (2010-2012)	Post-PCV13#1 (2013-2016)	Post-PCV13#2 (2017-2019)
Household income				
<$50,000				
Rate/100K PY (95% CI)	629.5 (551.6-718.4)	446.9 (392.7-508.5)	281.0 (246.6-320.3)	231.1 (177.4-301.0)
IRR (95% CI)	referent	0.71 (0.59-0.85)	0.45 (0.37-0.54)	0.37 (0.27-0.49)
No. of PY	34,948	51,469	80,064	23,799
$50,000-$99,999				
Rate/100K PY (95% CI)	472.5 (425.3-524.9)	389.0 (353.9-427.6)	243.5 (220.3-269.2)	189.8 (155.0-232.3)
IRR (95% CI)	referent	0.82 (0.71-0.95)	0.52 (0.45-0.60)	0.40 (0.32-0.50)
No. of PY	73,442	110,541	156,859	49,537
≥$100,000				
Rate/100K PY (95% CI)	331.1 (303.0-361.8)	287.7 (265.8-311.4)	201.6 (186.1-218.4)	183.0 (156.9-213.5)
IRR (95% CI)	referent	0.87 (0.77-0.98)	0.61 (0.54-0.69)	0.55 (0.46-0.66)
No. of PY	147,381	213,092	297,602	88,525
Unknown/missing				
Rate/100K PY (95% CI)	407.0 (379.1-437.0)	315.2 (289.2-343.5)	184.1 (165.5-204.8)	169.0 (155.2-184.2)
IRR (95% CI)	referent	0.77 (0.69-0.87)	0.45 (0.40-0.51)	0.42 (0.37-0.46)
No. of PY	186,736	164,683	184,670	308,786

AC-hPNE: all-cause hospitalized pneumonia; CI: confidence interval; IRR: incidence rate ratio; PCV13: 13-valent pneumococcal conjugate vaccine; PY: person year

vaccines

MDPI

Supplement - Table S2. Rates of AC-hPNA and disease incidence rate ratios among children aged 2-5 years by race, comorbidity, and household income

	Pre-PCV13	Peri-PCV13	Post-PCV13#1	Post-PCV13#2
Race				
Asian				
Rate/100K PY	225.5 (186.4-272.7)	220.9 (189.1-258.0)	161.8 (139.4-187.8)	115.7 (94.2-142.0)
IRR (95% CI)	referent	0.98 (0.77-1.25)	0.72 (0.56-0.91)	0.51 (0.39-0.68)
No. of PY	47,016	71,982	106,912	78,681
Black				
Rate/100K PY	329.3 (286.5-378.5)	284.6 (252.1-321.3)	204.3 (177.0-235.8)	126.8 (102.0-157.6)
IRR (95% CI)	referent	0.86 (0.72-1.04)	0.62 (0.51-0.76)	0.39 (0.30-0.50)
No. of PY	60,131	91,698	91,542	63,888
Hispanic				
Rate/100K PY	304.9 (268.9-345.6)	298.8 (268.5-332.6)	189.6 (168.4-213.4)	138.2 (118.7-160.9)
IRR (95% CI)	referent	0.98 (0.83-1.16)	0.62 (0.52-0.74)	0.45 (0.37-0.55)
No. of PY	80,030	112,436	144,509	120,107
White				
Rate/100K PY	256.0 (242.3-270.5)	216.3 (205.9-227.3)	172.6 (164.3-181.4)	126.1 (117.7-135.1)
IRR (95% CI)	referent	0.85 (0.78-0.91)	0.67 (0.63-0.73)	0.49 (0.45-0.54)
No. of PY	496,100	727,125	901,949	640,991
Unknown				
Rate/100K PY	263.6 (240.9-288.5)	247.4 (221.9-275.8)	177.9 (151.7-208.6)	142.6 (124.2-163.7)
IRR (95% CI)	referent	0.94 (0.81-1.08)	0.67 (0.56-0.81)	0.54 (0.46-0.64)
No. of PY	178,661	131,772	84,891	141,636
Comorbidity profile				
Low-risk				
Rate/100K PY	227.4 (217.4-238.0)	209.0 (200.6-217.8)	156.2 (149.5-163.2)	113.8 (107.5-120.6)
IRR (95% CI)	referent	0.92 (0.86-0.98)	0.69 (0.65-0.73)	0.50 (0.47-0.54)
No. of PY	825,731	1,097,027	1,289,814	1,015,417
At-risk				
Rate/100K PY	1,135.1 (1,030.5-1,250.4)	953.0 (859.7-1,056.4)	817.7 (733.7-911.3)	642.5 (557.7-740.1)
IRR (95% CI)	referent	0.84 (0.73-0.97)	0.72 (0.62-0.83)	0.57 (0.48-0.67)
No. of PY	36,207	37,987	39,989	29,885

AC-hPNE: all-cause hospitalized pneumonia; CI: confidence interval; IRR: incidence rate ratio; PCV13: 13-valent pneumococcal conjugate vaccine; PY: person year

Supplement - Table S2. Rates of AC-hPNA and disease incidence rate ratios among children aged 2-5 years by race, comorbidity, and household income (cont.)

	Pre-PCV13	Peri-PCV13	Post-PCV13#1	Post-PCV13#2
Household income				
<$50,000				
Rate/100K PY	372.9 (331.0-420.1)	312.9 (282.4-346.7)	215.2 (192.4-240.7)	151.1 (128.7-177.3)
IRR (95% CI)	referent	0.84 (0.72-0.98)	0.58 (0.49-0.68)	0.41 (0.33-0.49)
No. of PY	72,407	116,642	142,179	99,282
$50,000-$99,999				
Rate/100K PY	283.8 (258.3-311.8)	255.0 (235.5-276.0)	192.7 (177.3-209.4)	131.1 (116.5-147.5)
IRR (95% CI)	referent	0.90 (0.79-1.02)	0.68 (0.60-0.77)	0.46 (0.40-0.54)
No. of PY	152,920	240,032	288,008	210,519
≥$100,000				
Rate/100K PY	228.2 (212.8-244.7)	197.9 (186.1-210.4)	155.8 (146.5-165.8)	121.6 (111.6-132.5)
IRR (95% CI)	referent	0.87 (0.79-0.95)	0.68 (0.62-0.75)	0.53 (0.48-0.60)
No. of PY	343,583	513,923	642,976	425,943
Unknown/missing				
Rate/100K PY	273.4 (255.1-293.0)	250.0 (231.6-269.8)	186.6 (170.7-204.1)	130.5 (118.4-143.9)
IRR (95% CI)	referent	0.91 (0.83-1.01)	0.68 (0.61-0.76)	0.48 (0.42-0.54)
No. of PY	293,028	264,417	256,641	309,559

AC-hPNE: all-cause hospitalized pneumonia; CI: confidence interval; IRR: incidence rate ratio; PCV13: 13-valent pneumococcal conjugate vaccine; PY: person year

vaccines

Supplement - Table S3. Rates of AC-hPNA and disease incidence rate ratios among children aged 6-17 years by race, comorbidity profile, and household income

	Pre-PCV13	Peri-PCV13	Post-PCV13#1	Post-PCV13#2
Race				
Asian				
Rate/100K PY	58.2 (46.0-73.7)	44.0 (35.4-54.7)	28.8 (23.1-35.8)	25.0 (19.3-32.4)
IRR (95% CI)	referent	0.76 (0.55-1.04)	0.49 (0.36-0.68)	0.43 (0.30-0.61)
No. of PY	118,538	184,200	278,216	227,775
Black				
Rate/100K PY	72.8 (62.9-84.3)	56.7 (49.5-64.9)	37.2 (31.6-43.8)	28.7 (22.9-35.9)
IRR (95% CI)	referent	0.78 (0.64-0.95)	0.51 (0.41-0.64)	0.39 (0.30-0.51)
No. of PY	247,181	372,216	384,289	265,211
Hispanic				
Rate/100K PY	57.5 (49.2-67.2)	56.7 (49.7-64.7)	41.7 (36.6-47.6)	29.1 (24.5-34.5)
IRR (95% CI)	referent	0.99 (0.80-1.21)	0.72 (0.59-0.89)	0.51 (0.40-0.64)
No. of PY	274,700	391,563	532,509	454,158
White				
Rate/100K PY	63.0 (59.4-66.9)	53.1 (50.3-55.9)	40.5 (38.4-42.7)	30.8 (28.6-33.1)
IRR (95% CI)	referent	0.84 (0.78-0.91)	0.64 (0.59-0.70)	0.49 (0.44-0.54)
No. of PY	1,741,493	2,589,844	3,238,024	2,364,135
Unknown				
Rate/100K PY	61.9 (55.2-69.4)	64.3 (56.5-73.2)	39.5 (32.4-48.2)	31.0 (25.2-38.3)
IRR (95% CI)	referent	1.04 (0.87-1.23)	0.64 (0.51-0.80)	0.50 (0.39-0.64)
No. of PY	478,230	354,641	245,308	280,214
Comorbidity profile				
Low-risk				
Rate/100K PY	46.9 (44.4-49.6)	40.8 (38.8-42.9)	29.5 (27.9-31.1)	22.3 (20.8-24.0)
IRR (95% CI)	referent	0.87 (0.81-0.94)	0.63 (0.58-0.68)	0.48 (0.44-0.52)
No. of PY	2,731,647	3,700,928	4,460,844	3,433,479
At-risk				
Rate/100K PY	403.9 (370.6-440.2)	316.4 (292.2-342.6)	247.8 (227.8-269.6)	197.5 (176.7-220.6)
IRR (95% CI)	referent	0.78 (0.70-0.88)	0.61 (0.54-0.69)	0.49 (0.42-0.56)
No. of PY	128,496	191,535	217,502	158,014

AC-hPNE: all-cause hospitalized pneumonia; CI: confidence interval; IRR: incidence rate ratio; PCV13: 13-valent pneumococcal conjugate vaccine; PY: person year

Supplement - Table S3. Rates of AC-hPNA and disease incidence rate ratios among children aged 6-17 years by race, comorbidity profile, and household income (cont.)

	Pre-PCV13	Peri-PCV13	Post-PCV13#1	Post-PCV13#2
Household income				
<$50,000				
Rate/100K PY	73.3 (65.1-82.5)	56.6 (50.7-63.3)	37.9 (33.4-43.0)	30.2 (25.4-35.8)
IRR (95% CI)	referent	0.77 (0.66-0.91)	0.52 (0.44-0.62)	0.41 (0.33-0.51)
No. of PY	371,185	547,453	638,057	431,054
$50,000-$99,999				
Rate/100K PY	64.4 (58.2-71.2)	56.0 (51.3-61.1)	40.6 (36.9-44.6)	34.9 (30.9-39.4)
IRR (95% CI)	referent	0.87 (0.76-0.99)	0.63 (0.55-0.72)	0.54 (0.46-0.63)
No. of PY	591,908	897,860	1,030,612	733,941
≥$100,000				
Rate/100K PY	58.5 (54.1-63.3)	48.5 (45.3-51.9)	38.7 (36.2-41.3)	28.9 (26.4-31.6)
IRR (95% CI)	referent	0.83 (0.75-0.92)	0.66 (0.60-0.73)	0.49 (0.44-0.56)
No. of PY	1,076,923	1,727,208	2,264,909	1,663,471
Unknown/missing				
Rate/100K PY	63.2 (57.9-68.8)	64.7 (59.1-70.9)	42.6 (38.1-47.5)	27.9 (24.4-31.9)
IRR (95% CI)	referent	1.02 (0.90-1.16)	0.67 (0.59-0.77)	0.44 (0.38-0.52)
No. of PY	820,127	719,943	744,768	763,027

AC-hPNE: all-cause hospitalized pneumonia; CI: confidence interval; IRR: incidence rate ratio; PCV13: 13-valent pneumococcal conjugate vaccine; PY: person year
